# Mental Illness Diagnostic Criteria Can Be Simplified With Higher Symptom Prevalence and Correlations: A Simulation Study

**DOI:** 10.7759/cureus.85540

**Published:** 2025-06-07

**Authors:** Yi-Sheng Chao, Chao-Jung Wu

**Affiliations:** 1 Epidemiology and Public Health, Independent Researcher, Montreal, CAN; 2 Computer Science, Université du Québec à Montréal, Montreal, CAN

**Keywords:** correlations, decision trees, diagnostic and statistical manual of mental disorders (dsm), mental illness, prevalence, psychiatric symptoms

## Abstract

Introduction

The diagnostic criteria of mental illnesses have been found to assign excessive weights to certain input symptoms, and several input symptoms may not be significantly associated with their diagnosis. This study aims to investigate whether we can use input symptoms assigned much weight to diagnose mental illnesses with similar diagnostic accuracy as using all input symptoms.

Methods

The symptoms of three mental conditions were simulated based on a published study: major depressive episodes, dysthymic disorder, and manic episodes. We simulated symptoms with 0.05, 0.1, 0.3, 0.5, or 0.7 prevalence and 0, 0.1, 0.4, 0.7, or 0.9 correlations. For each of the 25 combinations of symptom prevalence and correlations, we simulated 100,000 subjects, and diagnoses were made based on the Diagnostic and Statistical Manual of Mental Disorders, 4th edition, text revision. For each simulation, we used a decision tree model that used a symptom with the best diagnostic accuracy to separate a population into diseased and non-diseased groups. This model continued using other symptoms to further separate the groups into subgroups. This process was repeated until the diagnostic accuracy could not be improved based on cross-validation errors. All analyses were implemented with R (v4.2.3; R Development Core Team, Vienna, Austria) and RStudio (v2023.6.0.421; RStudio Team, Boston, MA).

Results

The diagnoses of major depressive episodes, dysthymic disorder, and manic episodes required 15, 11, and 14 symptoms, respectively. There were opportunities to use fewer symptoms to approximate the diagnoses with 92% or higher sensitivities and specificities with certain combinations of symptom prevalence and correlations. For major depressive episodes, using two symptoms (“Depressed mood” and “Loss of interest or pleasure in daily activities” for more than two weeks) in the major criteria could diagnose the condition, with 100% sensitivity and specificity in some circumstances. Occasionally, the diagnosis of dysthymic disorder might be used to approximate the diagnosis of major depressive episodes.

Conclusion

There may lie opportunities to screen or follow up the diagnosis of major depressive episodes, dysthymic disorder, and manic episodes using fewer input symptoms, with at least 92% sensitivities and specificities. These opportunities exist in various combinations of symptom prevalence and correlations. However, there is a lack of real-world data on psychiatric symptoms and interventions to take advantage of these opportunities.

## Introduction

Mental illness diagnostic criteria have been found excessively complicated, and mental illness diagnoses may not be significantly associated with some of their input psychiatric symptoms. For example, some of the input psychiatric symptoms are not significantly associated with three of the disorders associated with the largest global mental illnesses burden: major depressive episodes, dysthymic disorder, and manic episodes [1,2]. The lack of significant associations between mental illnesses and their input symptoms results from the design of the diagnostic criteria that implicitly impose different weights to input symptoms [1]. Some of the input symptoms have been given so little weight that they do not contribute enough information to the diagnoses and lack significant associations with their diagnoses [2]. For example, input symptoms for major depressive episodes, dysthymic disorder, and manic episodes are grouped into major or minor criteria according to the Diagnostic and Statistical Manual of Mental Disorders, 4th edition, text revision (DSM-IV-TR) [1,2]. The input symptoms in the major criteria are often considered more important [2]. However, how much more weights are given to those in the major criteria is not declared or justified or explained by the DSM-IV-TR authors [2]. Some of the input symptoms classified as minor are given so little weight that they are not significantly associated with their diagnoses [3].

We know that input psychiatric symptoms collectively form the diagnoses. Assuming that the DSM-IV-TR criteria work well and correctly identify the cases with underlying pathologic causes to mental illnesses using psychiatric symptoms, many consider these diagnoses important targets for approximation. Researchers use many tools or questionnaires to approximate the diagnosis of mental illnesses, such as using the hypomania checklist and the mood disorder questionnaire to screen bipolar disorder [4]. Relatively few researchers study the diagnostic accuracy of individual psychiatric symptoms for mental illnesses [5].

Simplifying diagnostic criteria

Without sufficient evidence to assess the diagnostic accuracy of individual symptoms, we are unaware of how much individual psychiatric symptoms separately contribute to their diagnoses. If some symptoms contribute little to the diagnosis or lack significant associations with their diagnoses, we hypothesize that these symptoms can potentially be removed from the diagnostic criteria. We think current diagnostic approaches can be improved by simplifying the diagnostic criteria and reducing the number of input symptoms.

Interactions between diagnoses

Moreover, mental illness diagnoses can be correlated for several reasons, and the degree of their associations needs to be studied. For example, major depressive episodes and dysthymic disorder share six symptoms according to the DSM-IV-TR diagnostic criteria [1]. Some of the symptoms for major depressive episodes and manic episodes can be regarded as the same by certain individuals and lead to correlations between these two diagnoses [2]. The diagnostic criteria of mental illnesses have not been assessed for specificity [1] and may be subject to inferior specificity and overlap with other diagnoses. It is unclear whether there exist some symptoms that can be predictive of multiple diagnoses.

Due to unclear diagnostic accuracy of individual psychiatric symptoms and potential overlaps between diagnoses, this study aims to assess the role of individual psychiatric symptoms for diagnosing mental illnesses and explore the relationships between different diagnostic approaches using simulated data.

## Materials and methods

This is a diagnostic accuracy study using simulated cross-sectional data. For each simulation, 100,000 subjects were simulated with or without psychiatric symptoms. All of the simulated subjects were used for analysis. No other variables, such as demographic characteristics, were used.

Simulations

The simulations were conducted based on a previous study [1]. The presence of a symptom or a disorder was coded 1, while the absence of a symptom or a disorder was coded 0 [1]. The symptom prevalence was assumed to be 0.05, 0.1, 0.3, 0.5, or 0.7 for each simulation. The correlations between symptoms were assumed to be 0, 0.1, 0.4, 0.7, or 0.9 for each simulation. There were 25 combinations of symptom prevalence and between-symptom correlations. For each combination of symptom prevalence and correlations, 100,000 subjects were simulated. For each simulation, the symptoms were randomly assigned to the subjects based on the symptom prevalence and correlations. For example, with an assumed 0.1 prevalence and 0.3 between-symptom correlations, a symptom was randomly assigned to almost 10% of the simulated subjects, and this symptom correlated with other symptoms with a coefficient close to 0.3.

Diagnosing a disease involves classifying a population into diseased and non-diseased groups. We simulated populations with input psychiatric symptoms of different prevalence and correlations. We diagnosed three of the most common mood disorders in simulated populations: major depressive episodes (required for the diagnosis of major depressive disorder), dysthymic disorder, and manic episodes (required for the diagnosis of bipolar disorder) according to the DSM-IV-TR [1]. Table 1 lists the symptoms, intermediate variables, and bias variables for the diagnosis of the three conditions [1]. We assessed the diagnostic accuracy of the input symptoms for the classification of diagnoses. The DSM-IV-TR was used for simulation, instead of the newer DSM-5 [6], to align with previous simulation studies [1,7].

**Table 1:** Symptoms, intermediate variables, and bias variables for the diagnosis of major depressive episodes, dysthymic disorder, and manic episodes. Source: Ref [1]

The diagnoses that the variables belong to	Diagnoses, intermediate variables, bias variables, or symptoms	Variables	Definitions
‍‍‍‍‍Major depressive episodes for the diagnosis of major depressive disorder	Diagnosis	mde	Major depressive episodes
‍‍‍			Major criteria, essential for diagnosis: Depressed mood or a loss of interest or pleasure in daily activities for more than two weeks.
‍‍‍‍‍	Symptom	mde_ma1	Depressed mood for more than two weeks.
‍‍‍‍‍	Symptom	mde_ma2	Loss of interest or pleasure in daily activities for more than two weeks.
‍‍‍			Minor criteria (at least 5 of the symptoms/intermediate variables, including the two in major criteria)
‍‍‍‍‍	Intermediate variable	mde_mi3	Significant unintentional weight loss or gain
‍‍‍‍‍	Symptom	mde_mi3_1	Significant unintentional weight gain
‍‍‍‍‍	Symptom	mde_mi3_2	Significant unintentional weight loss
‍‍‍‍‍	Bias variable	mde_mi3_bias	Information about the intermediate variable not explained by the input symptoms
‍‍‍‍‍	Intermediate variable	mde_mi4	Insomnia or sleeping too much
‍‍‍‍‍	Symptom	mde_mi4_1	Insomnia
‍‍‍‍‍	Symptom	mde_mi4_2	Sleeping too much
‍‍‍‍‍	Bias variable	mde_mi4_bias	Information about the intermediate variable not explained by the input symptoms
‍‍‍‍‍	Intermediate variable	mde_mi5	Agitation or psychomotor retardation noticed by others
‍‍‍‍‍	Symptom	mde_mi5_1	Agitation
‍‍‍‍‍	Symptom	mde_mi5_2	Psychomotor retardation noticed by others
‍‍‍‍‍	Bias variable	mde_mi5_bias	Information about the intermediate variable not explained by the input symptoms
‍‍‍‍‍	Intermediate variable	mde_mi6	Fatigue or loss of energy
‍‍‍‍‍	Symptom	mde_mi6_1	Fatigue
‍‍‍‍‍	Symptom	mde_mi6_2	Loss of energy
‍‍‍‍‍	Bias variable	mde_mi6_bias	Information about the intermediate variable not explained by the input symptoms
‍‍‍‍‍	Intermediate variable	mde_mi7	Feelings of worthlessness or excessive guilt
‍‍‍‍‍	Symptom	mde_mi7_1	Feelings of worthlessness
‍‍‍‍‍	Symptom	mde_mi7_2	Feelings of excessive guilt
‍‍‍‍‍	Bias variable	mde_mi7_bias	Information about the intermediate variable not explained by the input symptoms
‍‍‍‍‍	Intermediate variable	mde_mi8	Diminished ability to think or concentrate, or indecisiveness
‍‍‍‍‍	Symptom	mde_mi8_1	Diminished ability to think or concentrate
‍‍‍‍‍	Symptom	mde_mi8_2	Indecisiveness
‍‍‍‍‍	Bias variable	mde_mi8_bias	Information about the intermediate variable not explained by the input symptoms
‍‍‍‍‍	Symptom	mde_mi9	Recurrent thoughts of death
‍‍‍			Information about the minor criteria not explained by the major or minor criteria
‍‍‍‍‍	Bias variable	mde_bias1	Information due to top censoring by choosing three intermediate variables in minor criteria
‍‍‍‍‍	Bias variable	mde_bias2	Information due to top censoring by choosing four intermediate variables in minor criteria
‍‍‍‍‍	Bias variable	mde_bias	Information about the diagnosis not explained by the intermediate variables
‍‍‍‍‍Dysthymic disorder	Diagnosis	dys	Dysthymic disorder
‍‍‍			Major criteria, essential for diagnosis
‍‍‍‍‍	Symptom	dys_ma	Depressed mood most of the day for more days than not, for at least 2 years
‍‍‍‍‍	Intermediate variable	dys_mi	Minor criteria (at least 2 of the following: dys_mi1, mde_mi4, mde_mi6, dys_mi4, mde_mi8, dys_mi6)
‍‍‍‍‍	Intermediate variable	dys_mi1	Poor appetite or overeating
‍‍‍‍‍	Symptom	dys_mi1_1	Poor appetite
‍‍‍‍‍	Symptom	dys_mi1_2	Overeating
‍‍‍‍‍	Bias variable	dys_mi1_bias	Information about the intermediate variable not explained by the input symptoms
‍‍‍‍‍	Intermediate variable	mde_mi4	Insomnia or sleeping too much
‍‍‍‍‍	Symptom	mde_mi4_1	Insomnia
‍‍‍‍‍	Symptom	mde_mi4_2	Sleeping too much
‍‍‍‍‍	Bias variable	mde_mi4_bias	Information about the intermediate variable not explained by the input symptoms
‍‍‍‍‍	Intermediate variable	mde_mi6	Low energy or fatigue
‍‍‍‍‍	Symptom	mde_mi6_1	Fatigue
‍‍‍‍‍	Symptom	mde_mi6_2	Loss of energy (low energy)
‍‍‍‍‍	Bias variable	mde_mi6_bias	Information about the intermediate variable not explained by the input symptoms
‍‍‍‍‍	Symptom	dys_mi4	Low self-esteem
‍‍‍‍‍	Intermediate variable	mde_mi8	Poor concentration or difficulty making decisions
‍‍‍‍‍	Symptom	mde_mi8_1	Diminished ability to think or concentrate (Poor concentration)
‍‍‍‍‍	Symptom	mde_mi8_2	difficulty making decisions (indecisiveness)
‍‍‍‍‍	Bias variable	mde_mi8_bias	Information about the intermediate variable not explained by the input symptoms
‍‍‍‍‍	Symptom	dys_mi6	Feelings of hopelessness
‍‍‍‍‍	Bias variable	dys_mi_bias	Information of minor criteria not explained by input variables
‍‍‍‍‍	Bias variable	dys_bias	Information of diagnosis not explained by major or minor criteria
‍‍‍‍‍Manic episodes for the diagnosis of bipolar disorder	Diagnosis	manic	Manic episodes
‍‍‍			Major criteria, essential for the diagnosis of a manic episode (more than one bipolar episode required to diagnose bipolar disorder): A distinct period of abnormally and persistently elevated, expansive, or irritable mood, lasting at least 1 week (or any duration if hospitalization is necessary)
‍‍‍‍‍	Symptom	man_ma1	Elevated mood, lasting at least 1 week
‍‍‍‍‍	Symptom	man_ma2	Expansive mood, lasting at least 1 week
‍‍‍‍‍	Symptom	man_ma3	Irritable mood, lasting at least 1 week
‍‍‍			Minor criteria (3 or more of the following have persisted; 4 if the mood is only irritable: man_mi1, man_mi2, man_mi3, man_mi4, man_mi5, man_mi6, man_mi7)
‍‍‍‍‍	Intermediate variable	man_mi1	Increased self-esteem or grandiosity
‍‍‍‍‍	Symptom	man_mi1_1	Increased self-esteem
‍‍‍‍‍	Symptom	man_mi1_2	Grandiosity
‍‍‍‍‍	Bias variable	man_mi1_bias	Information about the intermediate variable not explained by the input symptoms
‍‍‍‍‍	Symptom	man_mi2	Decreased need for sleep (e.g., feels rested after only 3 hours of sleep)
‍‍‍‍‍	Intermediate variable	man_mi3	More talkative than usual, or pressure to keep talking
‍‍‍‍‍	Symptom	man_mi3_1	More talkative than usual
‍‍‍‍‍	Symptom	man_mi3_2	Pressure to keep talking
‍‍‍‍‍	Bias variable	man_mi3_bias	Information about the intermediate variable not explained by the input symptoms
‍‍‍‍‍	Intermediate variable	man_mi4	Flight of ideas or subjective experience where thoughts are racing
‍‍‍‍‍	Symptom	man_mi4_1	Flight of ideas
‍‍‍‍‍	Symptom	man_mi4_2	Subjective experience that thoughts are racing
‍‍‍‍‍	Bias variable	man_mi4_bias	Information about the intermediate variable not explained by the input symptoms
‍‍‍‍‍	Symptom	man_mi5	Distractibility (i.e., attention too easily drawn to unimportant or irrelevant external stimuli)
‍‍‍‍‍	Intermediate variable	man_mi6	Increase in goal-directed activity (either socially, at work, or school, or sexually) or psychomotor agitation
‍‍‍‍‍	Symptom	man_mi6_1	Increase in goal-directed activity
‍‍‍‍‍	Symptom	man_mi6_2	Psychomotor agitation
‍‍‍‍‍	Bias variable	man_mi6_bias	Information about the intermediate variable not explained by the input symptoms
‍‍‍‍‍	Symptom	man_mi7	Excessive involvement in pleasurable activities that have a high potential for painful consequences (e.g., engaging in unrestrained buying sprees, sexual indiscretions, or foolish business investments)"
‍‍‍‍‍	Bias variable	man_bias1	Information about the diagnosis due to top-censoring for choosing at least three symptoms or intermediate variables
‍‍‍‍‍	Bias variable	man_bias2	Information about the diagnosis due to top-censoring for choosing at least four symptoms or intermediate variables
‍‍‍‍‍	Bias variable	man_bias	Information about the diagnosis not explained by symptoms

To mimic clinical practice, we thought that input symptoms that better distinguished those diseased from those not should first be investigated, regardless of the order of symptoms in the DSM criteria. This approach resembled decision tree models that screened for true cases using symptoms sequentially. We built decision trees for the diagnosis of mental illnesses using input psychiatric symptoms in an attempt to classify populations into diseased or non-diseased groups as accurately as possible. The decision trees began with an input psychiatric symptom that separated the population into two groups and resulted in diagnostic accuracy better than using the other input psychiatric symptoms [8]. From the symptom on top of the tree, there were arcs linked to subsequent symptoms that further separated the two subpopulations into different groups [8]. This process of separating subpopulations into two groups was repeated until the diagnostic accuracy could not be further improved [8]. Whether diagnostic accuracy could be improved was measured via 10-fold cross-validation errors [8]. Cross-validation separated the whole population into 10 groups of the same size, and the decision tree learned using nine groups was tested in the other group. Cross-validation was important because it reduced the chance of overfitting [9]. Overfitting occurred when the model that was developed in a population could lead to large prediction errors in other populations [9]. To avoid overfitting, cross-validation took a subset of the population to develop a model and obtain prediction errors by applying this model to the rest of the population [9]. When there was no further improvement in cross-validation errors, a decision tree was determined for a simulated population. Table 2 lists the equations to derive the diagnoses and intermediate variables [1], as well as the variables used for decision tree models. We constructed the decision trees via the rpart package [8] within the R environment (R Development Core Team, Vienna, Austria) [10,11].

**Table 2:** Disorders and their symptoms for diagnosis. The presence of a symptom or an intermediate variable or a diagnosis was coded 1, while the absence of a symptom or an intermediate variable or a diagnosis was coded 0. The definitions and names of the symptoms for the diagnosis of major depressive episodes, dysthymic disorder, and manic episodes according to the DSM-IV-TR are available in Table 1 and elsewhere [1].

Disorders	Equations	Input symptoms and intermediate variables	Bias variables	Variables used for decision tree models
Major depressive episodes (mde)	mde=mde_ma1 x mde_ma2 x (mde_mi3+ mde_mi4+ mde_mi5+mde_mi6+ mde_mi7+mde_mi8+ mde_mi9+mde_bias1) + (1- mde_ma1 x mde_ma2) x (me_ma1 x mde_ma2) x (mde_ mi3+mde_mi4+ mde_mi5+mde_mi6+ mde_mi7+mde_mi8+ mde_mi9+mde_bias2)	mde_ma1, mde_ma2, mde_mi3_1, mde_mi3_2, mde_mi3 (= mde_mi3_1 + mde_mi3_2 + mde_mi3_bias), mde_mi3_bias, mde_mi4_1, mde_mi4_2, mde_mi4 (= mde_mi4_1 + mde_mi4_2 + mde_mi4_bias), mde_mi4_bias, mde_mi5_1, mde_mi5_2, mde_mi5 (= mde_mi5_1 + mde_mi5_2 + mde_mi5_bias), mde_mi5_bias, mde_mi6_1, mde_mi6_2, mde_mi6 (= mde_mi6_1 + mde_mi6_2 + mde_mi6_bias), mde_mi6_bias, mde_mi7_1, mde_mi7_2, mde_mi7 (= mde_mi7_1 + mde_mi7_2 + mde_mi7_bias), mde_mi7_bias, mde_mi8_1, mde_mi8_2, mde_mi8 (= mde_mi8_1 + mde_mi8_2 + mde_mi8_bias), mde_mi8_bias, mde_mi9, mde_bias1, mde_bias2, mde_bias	mde_mi3_bias, mde_mi4_bias, mde_mi5_bias, mde_mi6_bias, mde_mi7_bias, mde_mi8_bias, mde_bias1, mde_bias2, mde_bias	mde_ma1, mde_ma2, mde_mi3_1, mde_mi3_2, mde_mi4_1, mde_mi4_2, mde_mi5_1, mde_mi5_2, mde_mi6_1, mde_mi6_2, mde_mi7_1, mde_mi7_2, mde_mi8_1, mde_mi8_2, mde_mi9, dys_ma, dys_mi1_1, dys_mi1_2, dys_mi4, dys_mi6, dys, man_ma1, man_ma2, man_ma3, man_mi1_1, man_mi1_2, man_mi2, man_mi3_1, man_mi3_2, man_mi4_1, man_mi4_2, man_mi5, man_mi6_1, man_mi6_2, man_mi7, manic
Dysthymic disorder (dys)	dys=dys_ma x dys_mi, where dys_mi = dys_mi1 + dys_mi2 + dys_mi3 + dys_mi4 + dys_mi5 + dys_mi6 + dys_mi_bias (mde and dys share 3 symptoms: dys_mi2 = mde_mi4, dys_mi3 = mde_mi6, and dys_mi5 = mde_mi8)	dys_ma, dys_mi1_1, dys_mi1_2, dys_mi1 (= dys_mi1_1 + dys_mi1_2 + dys_mi1_bias), dys_mi1_bias, mde_mi4 (= mde_mi4_1 + mde_mi4_2 + mde_mi4_bias), mde_mi4_1, mde_mi4_2, mde_mi4_bias, mde_mi6_1, mde_mi6_2, mde_mi6 (= mde_mi6_1 + mde_mi6_2 + mde_mi6_bias), mde_mi6_bias, dys_mi4, mde_mi8_1, mde_mi8_2, mde_mi8 (= mde_mi8_1 + mde_mi8_2 + mde_mi8_bias), mde_mi8_bias, dys_mi6, dys_mi, dys_mi_bias, dys_bias	dys_mi1_bias, mde_mi4_bias, mde_mi6_bias, mde_mi8_bias, dys_mi_bias, dys_bias	mde_ma1, mde_ma2, mde_mi3_1, mde_mi3_2, mde_mi4_1, mde_mi4_2, mde_mi5_1, mde_mi5_2, mde_mi6_1, mde_mi6_2, mde_mi7_1, mde_mi7_2, mde_mi8_1, mde_mi8_2, mde_mi9, mde, dys_ma, dys_mi1_1, dys_mi1_2, dys_mi4, dys_mi6, man_ma1, man_ma2, man_ma3, man_mi1_1, man_mi1_2, man_mi2, man_mi3_1, man_mi3_2, man_mi4_1, man_mi4_2, man_mi5, man_mi6_1, man_mi6_2, man_mi7, manic
Manic episodes (man)	man = (1- man_ma1 x man_ma2) x (man_ma1+man_ma2) x man_ma3 x (man_mi1+man_ mi2+ man_mi3+man_mi4+ man_mi5+man_mi6+ man_mi7+man_bias1) + (1 - (1 - man_ma1 x man_ma2)(man_ma1+ man_ma2)) x man_ma3 x (man_mi1+man_ mi2+ man_mi3+ man_mi4+man_mi5+ man_mi6+ man_mi7+man_bias2)	man_ma1, man_ma2, man_ma3, man_mi1_1, man_mi1_2, man_mi1 (= man_mi1_1 + man_mi1_2 + man_mi1_bias), man_mi1_bias, man_mi2, man_mi3_1, man_mi3_2, man_mi3 (= man_mi3_1 + man_mi3_2 + man_mi3_bias), man_mi3_bias, man_mi4_1, man_mi4_2, man_mi4 (= man_mi4_1 + man_mi4_2 + man_mi4_bias), man_mi4_bias, man_mi5, man_mi6_1, man_mi6_2, man_mi6 (= man_mi6_1 + man_mi6_2 + man_mi6_bias), man_mi6_bias, man_mi7, man_bias1, man_bias2, man_bias	man_mi1_bias, man_mi3_bias, man_mi4_bias, man_mi6_bias, man_bias1, man_bias2, man_bias	mde_ma1, mde_ma2, mde_mi3_1, mde_mi3_2, mde_mi4_1, mde_mi4_2, mde_mi5_1, mde_mi5_2, mde_mi6_1, mde_mi6_2, mde_mi7_1, mde_mi7_2, mde_mi8_1, mde_mi8_2, mde_mi9, mde, dys_ma, dys_mi1_1, dys_mi1_2, dys_mi4, dys_mi6, dys, man_ma1, man_ma2, man_ma3, man_mi1_1, man_mi1_2, man_mi2, man_mi3_1, man_mi3_2, man_mi4_1, man_mi4_2, man_mi5, man_mi6_1, man_mi6_2, man_mi7

Correlations

For each combination of symptom prevalence and between-symptom correlations, the simulations were repeated 100 times to derive the average statistics. After each simulation, we obtained the Pearson’s correlation coefficients for the correlations between disorders and input symptoms and between symptoms [7].

Decision tree statistics

The outcomes for each decision tree were the symptoms used to diagnose three mood disorders (major depressive episodes, dysthymic disorder, and manic episodes) and the sensitivities and specificities of the decision trees for diagnosing the disorders. For each combination of symptom prevalence and between-symptom correlations, the symptoms used for approximating the disorders, sensitivities, and specificities were summarized from 100 simulations. The simulations and analyses were conducted within the R environment (v4.2.3; R Development Core Team, Vienna, Austria) [10] and RStudio (v2023.6.0.421; RStudio Team, Boston, MA) [11].

## Results

The three disorders were diagnosed according to the DSM-IV-TR. Following the steps in a previous article, the symptoms were simulated with prevalence and correlations that were similar to assumed values [1]. This suggested that the simulations were successfully implemented.

Decision trees

**Figure 1:**
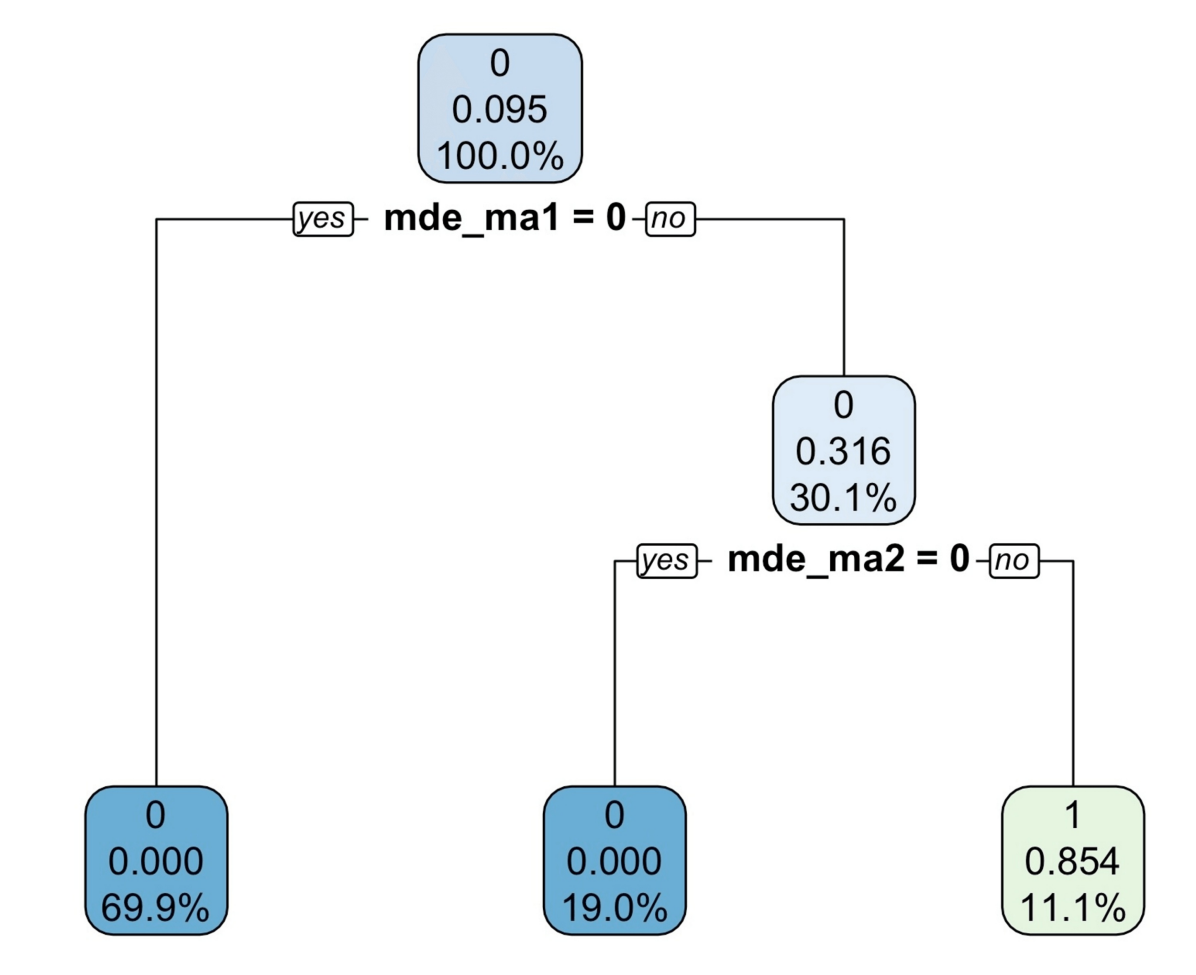
The diagnosis of major depressive disorder using its input symptoms assuming 0.3 symptom prevalence and 0.1 correlations between symptoms. Assuming 0.3 symptom prevalence and 0.1 correlations between symptoms in a simulated population of 100,000 subjects. In the decision tree, the confirmation of two symptoms in the major criteria (mde_ma1: Depressed mood for more than two weeks, and mde_ma2: Loss of interest or pleasure in daily activities for more than two weeks) achieved an overall sensitivity and specificity of 100% and 98.2% for the diagnosis of major depressive episodes, respectively. The values at the top, in the middle, and at the bottom of each box are the predicted values (disorder status for the simulated subjects; 0 = no diagnosis; 1 = with a diagnosis), mean values (prevalence of the diagnosis in the subpopulation), and the proportions of the population, respectively.

For each diagnosis in a simulated population, we used eligible symptoms to build a decision tree. Figure 1 shows a decision tree obtained using symptoms to diagnose major depressive episodes in a simulation, with symptom prevalence close to 0.3 and correlations close to 0.1 between symptoms. In the decision tree, each node included three numbers. Predicted values according to the most prevalent values (disorder status for the simulated subjects; 0 = no disorder; 1 = with a disorder), prevalence of the disorder, and population proportions were on the top, in the middle, and at the bottom, respectively.

In detail, the first node indicated that 0.093 of the population was diagnosed with major depressive episodes, denoted by mde, while 70% of the population did not have the first symptom in the major criteria, mde_ma1 (Depressed mood for more than two weeks), and this matched the assumed values. Those with the symptom, mde_ma1, were then screened with the second symptom in the major criteria, mde_ma2 (Loss of interest or pleasure in daily activities for more than two weeks). Additionally, 10.9% of the population had both symptoms in the major criteria, and 0.854 or 85.4% of those with these two symptoms were diagnosed with major depressive episodes (according to all eligible symptoms). This led to an overall sensitivity and specificity of 100% and 98.2%, respectively, for the diagnosis of major depressive episodes using two of the input symptoms.

Diagnosis approximation using input symptoms of its own

Major Depressive Episodes

The diagnosis of major depressive episodes requires 15 input symptoms according to the DSM-IV-TR criteria, 12 of which formed six intermediate variables and six bias variables to diagnose this disorder. When the symptoms occurred among nearly 5% of the population (assumed prevalence = 0.05) and the input symptoms were uncorrelated (assumed correlation = 0), the disorder was not observed in the population (diagnosis prevalence = 0). This could be found in Table 3, in which there were 500 simulations and only 400 simulations with any diagnosis, with assumed symptom prevalence = 0.05 or assumed between-symptom correlation = 0.1. Overall, using two to 15 symptoms to approximate the diagnosis could achieve an average sensitivity and specificity of 0.995 and 0.967, respectively. The minimal sensitivity was at least 0.975 among all simulations. The minimal specificity was 0.339 when symptom prevalence was close to 0.1 and between-symptom correlation was close to 0.

**Table 3:** Summary of the decision trees for the diagnosis of major depressive episodes. sen/spe = sensitivity or specificity of diagnosing major depressive episodes using decision trees. The definitions and names of the symptoms for the diagnosis of major depressive episodes according to the Diagnostic and Statistical Manual of Mental Disorders, 4th edition, text revision, are available in Table 1.

Variables	Prevalence=0.05	Prevalence=0.1	Prevalence=0.3	Prevalence=0.5	Prevalence=0.7	Correlation=0	Correlation=0.1	Correlation=0.4	Correlation=0.7	Correlation=0.9
Number of simulations	500	500	500	500	500	500	500	500	500	500
Number of simulations with any diagnosis	400	500	500	500	500	400	500	500	500	500
Average number of variables for prediction	4.93	5.21	3.02	2	2	5.43	5.45	2.38	2	2
Max number of variables for prediction	15	14	7	2	2	13	15	7	2	2
Min number of variables for prediction	2	2	2	2	2	2	2	2	2	2
Median number of variables for prediction	2	2	2	2	2	2	2	2	2	2
Average sensitivities	0.999	0.998	0.991	0.992	0.995	0.995	0.993	0.993	0.996	0.998
Average specificities	0.967	0.883	0.986	1	1	0.862	0.955	0.997	1	1
Max sensitivities	1	1	0.998	0.998	1	1	1	0.999	0.999	1
Max specificities	1	1	1	1	1	1	1	1	1	1
Min sensitivities	0.998	0.996	0.975	0.988	0.99	0.975	0.981	0.989	0.992	0.995
Min specificities	0.802	0.339	0.921	1	1	0.339	0.802	0.965	1	1
Proportion of simulations with >0.99 sen/spe	0.506	0.6	0.598	0.63	1	0.4	0.2	0.734	1	1
Proportion of simulations with >0.98 sen/spe	0.512	0.6	0.792	1	1	0.4	0.592	0.912	1	1
Proportion of simulations with >0.97 sen/spe	0.558	0.6	0.796	1	1	0.404	0.592	0.958	1	1
Proportion of simulations with >0.96 sen/spe	0.6	0.6	0.804	1	1	0.404	0.6	1	1	1
Proportion of simulations with >0.95 sen/spe	0.6	0.6	0.804	1	1	0.404	0.6	1	1	1
Proportion of simulations with >0.90 sen/spe	0.644	0.678	1	1	1	0.6	0.722	1	1	1
Proportion of simulations with >0.85 sen/spe	0.778	0.794	1	1	1	0.6	0.972	1	1	1
Proportion of simulations with >0.80 sen/spe	0.8	0.8	1	1	1	0.6	1	1	1	1
Proportion of simulations with >0.75 sen/spe	0.8	0.8	1	1	1	0.6	1	1	1	1
Symptoms Used Below, Frequencies										
mde_ma1	400	500	500	500	500	400	500	500	500	500
mde_ma2	400	500	500	500	500	400	500	500	500	500
mde_mi3_1	85	117	39	0	0	104	122	15	0	0
mde_mi3_2	91	113	39	0	0	98	132	13	0	0
mde_mi4_1	82	125	33	0	0	100	131	9	0	0
mde_mi4_2	92	122	33	0	0	100	137	10	0	0
mde_mi5_1	91	116	39	0	0	102	130	14	0	0
mde_mi5_2	91	123	39	0	0	103	137	13	0	0
mde_mi6_1	83	120	34	0	0	101	123	13	0	0
mde_mi6_2	87	123	34	0	0	97	132	15	0	0
mde_mi7_1	82	114	33	0	0	95	123	11	0	0
mde_mi7_2	87	115	33	0	0	101	122	12	0	0
mde_mi8_1	83	124	26	0	0	94	123	16	0	0
mde_mi8_2	97	120	26	0	0	94	132	17	0	0
mde_mi9	120	175	102	0	0	184	180	33	0	0

When the prevalence of symptoms reached 0.5 or higher or when the between-symptom correlations were 0.7 or higher, it only required two symptoms to achieve a sensitivity of at least 0.988 and a perfect specificity (1.0). In Table 3, the two symptoms used were mde_ma1 (Depressed mood for more than two weeks) and mde_ma2 (Loss of interest or pleasure in daily activities for more than two weeks) (the 2 symptoms in the major criteria for the diagnosis of major depressive episodes) that were used 500 times in the 500 simulations with assumed symptom prevalence = 0.5 or 0.7 or with assumed between-symptom correlations = 0.7 or 0.9.

In Figure 1, on top of the decision tree, 70% of the population was excluded from the diagnosis of mde (major depressive episodes) because they did not have the symptom, mde_ma1 (Depressed mood for more than two weeks) = 0. Among 30% of the population with the symptom, mde_ma1 = 1, 10.9% of the population was considered to have the diagnosis of mde (major depressive episodes), but only part (0.85 or 85%) actually had the diagnosis of mde (major depressive episodes). This led to an overall sensitivity and specificity of 100% and 98.2%, respectively. In this case, using two of the 15 input symptoms to diagnose major depressive episodes could lead to a 100% sensitivity and a 98.2% specificity.

There were opportunities to simplify the diagnostic criteria of major depressive episodes based on the diagnostic accuracy. When symptom prevalence was 0.5 or higher or between-symptom correlations were 0.7 or higher, the sensitivity or specificity of decision trees reached 0.98 or higher using 2 input symptoms: mde_ma1 (Depressed mood for more than two weeks) and mde_ma2 (Loss of interest or pleasure in daily activities for more than two weeks). When symptom prevalence was 0.3 or higher and between-symptom correlations were 0.1 or higher, the sensitivity or specificity of decision trees reached 0.96 or higher using these two input symptoms. When symptom prevalence was 0.1 or higher and between-symptom correlations were 0.4 or higher, the sensitivity or specificity of decision trees reached 0.98 or higher using these two input symptoms.

Dysthymic Disorder

The diagnosis of dysthymic disorder required 11 input symptoms, 8 of which formed 4 intermediate variables and 4 bias variables for the diagnosis. Six of the input symptoms were also used for the diagnosis of major depressive episodes (in Table 1, or refer to previous research for details [1]). The diagnosis of dysthymic disorder could occur in all simulations in Table 4. Using 1 to 11 input symptoms to diagnose dysthymic disorder, the average sensitivity and specificity were 0.982 for both. The minimal sensitivity was 0.930, assuming symptom prevalence as 0.3 and between-symptom correlations as 0. The minimal specificity was 0.807, assuming symptom prevalence as 0.05 and between-symptom correlations as 0. It only required 1 input symptom (dys_ma: Depressed mood most of the day for more days than not, for at least 2 years) to achieve a perfect sensitivity (100%) and a specificity of at least 0.952, assuming symptom prevalence as 0.5 or 0.7 or assuming between-symptom correlations as 0.9. The symptom in the major criteria, dys_ma (Depressed mood most of the day for more days than not, for at least two years), was used in all simulations to approximate the diagnosis of dysthymic disorder.

**Table 4:** Summary of the decision trees for the diagnosis of dysthymic disorder. sen/spe = sensitivity or specificity of diagnosing dysthymic disorder using decision trees. The definitions and names of the symptoms for the diagnosis of major depressive episodes and dysthymic disorder according to the DSM-IV-TR are available in Table 1.

Variables	Prevalence=0.05	Prevalence=0.1	Prevalence=0.3	Prevalence=0.5	Prevalence=0.7	Correlation=0	Correlation=0.1	Correlation=0.4	Correlation=0.7	Correlation=0.9
Number of simulations	500	500	500	500	500	500	500	500	500	500
Number of simulations with any diagnosis	500	500	500	500	500	500	500	500	500	500
Average number of variables for prediction	6.57	5.88	2.46	1	1	5.01	5.26	4	1.63	1
Max number of variables for prediction	11	11	7	1	1	11	11	7	5	1
Min number of variables for prediction	1	1	1	1	1	1	1	1	1	1
Median number of variables for prediction	6	6	1	1	1	1	1	6	1	1
Average sensitivities	0.998	0.994	0.967	0.973	0.98	0.983	0.978	0.979	0.982	0.99
Average specificities	0.946	0.969	0.993	1	1	0.969	0.963	0.981	0.996	1
Max sensitivities	1	1	0.991	0.987	1	1	0.999	0.997	0.999	0.998
Max specificities	1	1	1	1	1	1	1	1	1	1
Min sensitivities	0.994	0.988	0.93	0.952	0.959	0.93	0.931	0.952	0.965	0.98
Min specificities	0.807	0.891	0.964	1	1	0.807	0.861	0.935	0.971	1
Proportion of simulations with >0.99 sen/spe	0.254	0.406	0.186	0	0.338	0.218	0.138	0	0.242	0.586
Proportion of simulations with >0.98 sen/spe	0.256	0.406	0.202	0.4	0.598	0.418	0.2	0.002	0.244	0.998
Proportion of simulations with >0.97 sen/spe	0.402	0.452	0.672	0.596	0.614	0.452	0.272	0.202	0.81	1
Proportion of simulations with >0.96 sen/spe	0.568	0.77	0.676	0.8	0.996	0.576	0.478	0.756	1	1
Proportion of simulations with >0.95 sen/spe	0.606	0.79	0.676	1	1	0.584	0.49	0.998	1	1
Proportion of simulations with >0.90 sen/spe	0.766	0.962	1	1	1	0.85	0.878	1	1	1
Proportion of simulations with >0.85 sen/spe	0.956	1	1	1	1	0.956	1	1	1	1
Proportion of simulations with >0.80 sen/spe	1	1	1	1	1	1	1	1	1	1
Proportion of simulations with >0.75 sen/spe	1	1	1	1	1	1	1	1	1	1
Variables used below, frequencies										
mde_mi4_1	265	221	62	0	0	200	209	113	26	0
mde_mi4_2	266	229	59	0	0	200	210	118	26	0
mde_mi6_1	270	241	62	0	0	201	215	125	32	0
mde_mi6_2	263	233	59	0	0	201	208	119	27	0
mde_mi8_1	263	227	50	0	0	200	198	115	27	0
mde_mi8_2	268	226	54	0	0	200	205	116	27	0
dys_ma	500	500	500	500	500	500	500	500	500	500
dys_mi1_1	258	226	57	0	0	201	200	116	24	0
dys_mi1_2	261	240	49	0	0	201	210	121	18	0
dys_mi4	332	299	138	0	0	201	237	278	53	0
dys_mi6	337	298	138	0	0	201	237	279	56	0

There were opportunities to simplify the diagnostic criteria of dysthymic disorder using one input symptom, dys_ma (Depressed mood most of the day for more days than not, for at least 2 years), based on the diagnostic accuracy. When symptom prevalence was 0.5 or higher or between-symptom correlations were 0.9, the sensitivity or specificity of decision trees reached 0.95 or higher using this input symptom. When symptom prevalence was 0.3 and between-symptom correlations were 0 or 0.7, the sensitivity or specificity of decision trees reached 0.92 or higher using the same input symptom.

Manic Episodes

The diagnosis of manic episodes required 14 input symptoms, eight of which produced four intermediate variables and four bias variables. The input symptoms of manic episodes were not correlated with the input symptoms of the other disorders. The diagnosis of manic episodes did not appear in some simulations, especially when assumed between-symptom correlations equalled 0 and assumed symptom prevalence was 0.1 or lower. Using 1-13 input symptoms to approximate the diagnosis could achieve an average sensitivity and specificity of 0.982 and 0.908, respectively (Table 5). The minimal sensitivity was 0.868, assuming symptom prevalence as 0.5 and between-symptom correlations as 0.1. The minimal specificity was 0.101, assuming symptom prevalence as 0.1 and between-symptom correlations as 0. When only one input symptom was required to approximate the diagnosis of manic episodes in Table 5, this symptom was the third symptom in the major criteria, man_ma3 (Irritable mood, lasting at least one week), because the diagnostic criteria unintentionally placed too much weight on this symptom [1,2].

**Table 5:** Summary of the decision trees for the diagnosis of manic episodes. sen/spe = sensitivity or specificity of diagnosing manic episodes using decision trees. The definitions and names of the symptoms for the diagnosis of manic episodes according to the DSM-IV-TR are available in Table 1.

Variables	Prevalence=0.05	Prevalence=0.1	Prevalence=0.3	Prevalence=0.5	Prevalence=0.7	Correlation=0	Correlation=0.1	Correlation=0.4	Correlation=0.7	Correlation=0.9
Number of simulations	500	500	500	500	500	500	500	500	500	500
Number of simulations with any diagnosis	402	467	500	500	500	369	500	500	500	500
Average number of variables for prediction	6.19	5.83	5.06	2.12	1.12	4.56	5.9	4.65	2.85	1.96
Max number of variables for prediction	13	13	10	4	4	13	13	11	5	4
Min number of variables for prediction	3	3	1	1	1	1	1	1	1	1
Median number of variables for prediction	4.5	6	4	1	1	1	7	4	4	1
Average sensitivities	0.998	0.995	0.97	0.936	0.942	0.952	0.963	0.962	0.97	0.982
Average specificities	0.867	0.785	0.904	0.985	1	0.778	0.81	0.955	0.988	0.992
Max sensitivities	1	1	0.986	0.977	0.977	1	0.999	0.998	0.998	0.999
Max specificities	0.992	0.992	1	1	1	1	1	1	1	1
Min sensitivities	0.995	0.989	0.957	0.868	0.896	0.882	0.868	0.896	0.927	0.959
Min specificities	0.308	0.101	0.719	0.942	0.986	0.101	0.532	0.881	0.968	0.966
Proportion of simulations with >0.99 sen/spe	0.024	0.036	0	0	0	0	0	0	0	0.06
Proportion of simulations with >0.98 sen/spe	0.154	0.12	0.396	0	0	0	0	0	0.318	0.352
Proportion of simulations with >0.97 sen/spe	0.38	0.398	0.4	0.188	0.214	0.2	0	0	0.614	0.766
Proportion of simulations with >0.96 sen/spe	0.4	0.4	0.5	0.202	0.412	0.2	0	0.1	0.616	0.998
Proportion of simulations with >0.95 sen/spe	0.4	0.4	0.6	0.402	0.44	0.2	0	0.426	0.616	1
Proportion of simulations with >0.90 sen/spe	0.55	0.596	0.6	0.772	0.838	0.2	0.372	0.784	1	1
Proportion of simulations with >0.85 sen/spe	0.6	0.6	0.62	1	1	0.4	0.42	1	1	1
Proportion of simulations with >0.80 sen/spe	0.6	0.6	0.8	1	1	0.4	0.6	1	1	1
Proportion of simulations with >0.75 sen/spe	0.6	0.6	0.8	1	1	0.4	0.6	1	1	1
Symptoms used below, frequencies										
man_ma1	24	34	0	0	0	33	25	0	0	0
man_ma2	28	30	0	0	0	30	27	1	0	0
man_ma3	402	467	500	500	500	369	500	500	500	500
man_mi1_1	128	118	106	0	0	103	158	71	9	11
man_mi1_2	134	123	106	0	0	104	156	78	9	16
man_mi2	344	426	400	187	20	165	382	411	288	131
man_mi3_1	133	121	107	0	0	103	166	72	6	14
man_mi3_2	121	116	107	0	0	102	153	71	6	12
man_mi4_1	132	101	107	0	0	92	158	70	12	8
man_mi4_2	128	113	107	0	0	93	160	75	7	13
man_mi5	347	418	400	187	20	155	385	412	287	133
man_mi6_1	115	114	95	0	0	86	151	78	6	3
man_mi6_2	115	126	95	0	0	95	148	76	12	5
man_mi7	339	417	400	187	20	153	383	412	283	132

When assumed symptom prevalence was 0.3 and assumed between-symptom correlations were close to 0.9, it took man_ma3 (Irritable mood, lasting at least 1 week) to approximate the diagnosis with an average sensitivity and specificity of 0.981 and 1, respectively. When assumed symptom prevalence was 0.5 and assumed between-symptom correlations were 0 or 0.9, it took man_ma3 to approximate the diagnosis with an average sensitivity of 0.887 and 0.971, respectively. When assumed symptom prevalence was 0.7 and assumed between-symptom correlations were 0 or 0.1, or 0.9, it took man_ma3 to approximate the diagnosis with an average sensitivity of 0.975, 0.935, and 0.962, respectively.

There were opportunities to simplify the diagnostic criteria of manic episodes based on the diagnostic accuracy. When assumed symptom prevalence was 0.3 or higher and assumed between-symptom correlations were 0.9, the sensitivity or specificity of decision trees reached 0.95 or higher using one input symptom: man_ma3 (Irritable mood, lasting at least one week). When assumed symptom prevalence was 0.7 and assumed between-symptom correlations were 0, 0.1, or 0.4, the sensitivity or specificity of decision trees reached 0.92 or higher using at most four input symptoms: man_ma3 (Irritable mood, lasting at least one week), man_mi2 (Decreased need for sleep), man_mi5 (Distractibility), and man_mi7 (Excessive involvement in pleasurable activities that have a high potential for painful consequences).

Diagnosis approximation using the symptoms of its own and those of other disorders

Major Depressive Episodes

When approximating the diagnosis of major depressive episodes using the input symptoms of its own and the other disorders, the decision trees might differ from those only using its own input symptoms. There were several differences. There were two diagnoses (dysthymic disorder and manic episodes), and 19 input symptoms of these two diagnoses were used to approximate the diagnosis of major depressive episodes (Table 6). This led to more variables being eligible and used in many decision trees. However, the diagnostic accuracy of decision trees might not change with more symptoms eligible for approximation. Similar to the approximation using its own input symptoms, when symptom prevalence reached 0.5 or 0.7 or when between-symptom correlations were 0.7 or 0.9, it only required the same two input symptoms in the major criteria (mde_ma1: Depressed mood for more than two weeks; and mde_ma2: Loss of interest or pleasure in daily activities for more than two weeks) to approximate the diagnosis with nearly perfect diagnostic accuracy (sensitivity specificity > 0.98 for all). The opportunities to simplify the diagnostic criteria of major depressive episodes based on the diagnostic accuracy did not change while including the input symptoms of the other disorders.

**Table 6:** Summary of the decision trees for the diagnosis of major depressive episodes using the input symptoms of its own and the other disorders. sen/spe = sensitivity or specificity of diagnosing major depressive episodes using decision trees. The definitions and names of the symptoms for the diagnosis of major depressive episodes, dysthymic disorder, and manic episodes according to the DSM-IV-TR are available in Table 1.

Variables	Prevalence=0.05	Prevalence=0.1	Prevalence=0.3	Prevalence=0.5	Prevalence=0.7	Correlation=0	Correlation=0.1	Correlation=0.4	Correlation=0.7	Correlation=0.9
Number of simulations	500	500	500	500	500	500	500	500	500	500
Number of simulations with any diagnosis	400	500	500	500	500	400	500	500	500	500
Average number of variables for prediction	4.86	5.21	3.01	2	2	5.36	5.52	2.3	2	2
Max number of variables for prediction	15	14	7	2	2	13	15	7	2	2
Min number of variables for prediction	2	2	2	2	2	2	2	2	2	2
Median number of variables for prediction	2	2	2	2	2	2	2	2	2	2
Average sensitivities	0.999	0.998	0.991	0.992	0.995	0.995	0.993	0.993	0.996	0.998
Average specificities	0.967	0.879	0.986	1	1	0.859	0.954	0.998	1	1
Max sensitivities	1	1	0.998	0.998	1	1	1	0.999	0.999	1
Max specificities	1	1	1	1	1	1	1	1	1	1
Min sensitivities	0.998	0.996	0.975	0.988	0.99	0.975	0.981	0.989	0.992	0.995
Min specificities	0.793	0.346	0.922	1	1	0.346	0.793	0.962	1	1
Proportion of simulations with >0.99 sen/spe	0.526	0.6	0.596	0.634	1	0.4	0.2	0.756	1	1
Proportion of simulations with >0.98 sen/spe	0.532	0.6	0.788	1	1	0.4	0.588	0.932	1	1
Proportion of simulations with >0.97 sen/spe	0.578	0.6	0.798	1	1	0.41	0.588	0.978	1	1
Proportion of simulations with >0.96 sen/spe	0.6	0.6	0.81	1	1	0.41	0.6	1	1	1
Proportion of simulations with >0.95 sen/spe	0.6	0.602	0.81	1	1	0.41	0.602	1	1	1
Proportion of simulations with >0.90 sen/spe	0.644	0.684	1	1	1	0.6	0.728	1	1	1
Proportion of simulations with >0.85 sen/spe	0.774	0.798	1	1	1	0.6	0.972	1	1	1
Proportion of simulations with >0.80 sen/spe	0.798	0.8	1	1	1	0.6	0.998	1	1	1
Proportion of simulations with >0.75 sen/spe	0.8	0.8	1	1	1	0.6	1	1	1	1
Symptoms and diagnoses used below										
mde_ma1	400	500	500	500	500	400	500	500	500	500
mde_ma2	400	500	500	500	500	400	500	500	500	500
mde_mi3_1	83	120	32	0	0	96	135	4	0	0
mde_mi3_2	81	122	32	0	0	93	136	6	0	0
mde_mi4_1	81	126	35	0	0	108	128	6	0	0
mde_mi4_2	74	109	35	0	0	99	113	6	0	0
mde_mi5_1	92	129	36	0	0	109	132	16	0	0
mde_mi5_2	80	115	36	0	0	94	123	14	0	0
mde_mi6_1	93	124	40	0	0	98	146	13	0	0
mde_mi6_2	85	130	40	0	0	106	135	14	0	0
mde_mi7_1	87	114	27	0	0	88	126	14	0	0
mde_mi7_2	84	101	27	0	0	79	121	12	0	0
mde_mi8_1	89	118	32	0	0	100	126	13	0	0
mde_mi8_2	82	121	32	0	0	92	133	10	0	0
mde_mi9	110	166	101	0	0	175	179	23	0	0
dys_ma	0	1	0	0	0	1	0	0	0	0
dys_mi1_1	4	0	0	0	0	0	4	0	0	0
dys_mi1_2	2	0	0	0	0	0	2	0	0	0
dys_mi4	3	1	0	0	0	0	4	0	0	0
dys_mi6	2	0	0	0	0	0	2	0	0	0
dys	13	2	0	0	0	0	15	0	0	0
man_mi2	0	2	0	0	0	2	0	0	0	0
man_mi3_1	0	1	0	0	0	1	0	0	0	0
man_mi4_2	0	1	0	0	0	1	0	0	0	0

In the decision trees, we could observe the interactions between diagnoses. The diagnosis of dysthymic disorder was occasionally used to approximate the diagnosis of major depressive episodes, when the input symptoms had prevalence close to or less than 0.1 and between-symptom correlations similar to 0.1.

Dysthymic Disorders

No other diagnoses or the symptoms of the other diagnoses were included in the decision trees, even the other diagnoses and their symptoms were eligible for the inclusion in the decision trees for approximating the diagnosis of dysthymic disorder.

Manic Episodes

Although the input symptoms of manic episodes were not correlated with those of the other two disorders, there was a chance that the input symptoms of the other disorders were used for approximating the diagnosis of manic episodes (Table 7). However, there might be fewer opportunities to simplify the diagnostic criteria while considering the input symptoms of the other two diagnoses in decision trees. Only when between-symptom correlations reached 0.7 or 0.9 did it take one to four input symptoms to achieve at least 90% of sensitivities or specificities.

**Table 7:** Summary of the decision trees for the diagnosis of manic episodes using the input symptoms of its own and the other disorders. sen/spe = sensitivity or specificity of diagnosing manic episodes using decision trees. The definitions and names of the symptoms for the diagnosis of major depressive episodes, dysthymic disorder, and manic episodes according to the DSM-IV-TR are available in Table 1.

Variables	Prevalence=0.05	Prevalence=0.1	Prevalence=0.3	Prevalence=0.5	Prevalence=0.7	Correlation=0	Correlation=0.1	Correlation=0.4	Correlation=0.7	Correlation=0.9
Number of simulations	500	500	500	500	500	500	500	500	500	500
Number of simulations with any diagnosis	401	450	500	500	500	351	500	500	500	500
Average number of variables for prediction	6.26	5.73	5.01	2.11	1.17	4.39	5.89	4.67	2.85	1.98
Max number of variables for prediction	14	13	10	4	4	13	14	10	4	4
Min number of variables for prediction	3	1	1	1	1	1	1	1	1	1
Median number of variables for prediction	4	6	4	1	1	1	7	4	4	1
Average sensitivities	0.998	0.995	0.97	0.936	0.943	0.949	0.963	0.963	0.97	0.982
Average specificities	0.87	0.808	0.904	0.985	0.999	0.808	0.811	0.954	0.988	0.992
Max sensitivities	1	1	0.986	0.973	0.977	1	0.999	0.998	0.997	0.999
Max specificities	0.991	1	1	1	1	1	1	1	1	1
Min sensitivities	0.994	0.989	0.957	0.868	0.896	0.883	0.868	0.896	0.928	0.958
Min specificities	0.333	0.13	0.72	0.942	0.986	0.13	0.534	0.87	0.947	0.968
Proportion of simulations with >0.99 sen/spe	0.026	0.04	0	0	0	0	0	0	0	0.066
Proportion of simulations with >0.98 sen/spe	0.178	0.124	0.396	0	0	0	0	0	0.314	0.384
Proportion of simulations with >0.97 sen/spe	0.384	0.396	0.4	0.19	0.216	0.2	0	0	0.614	0.772
Proportion of simulations with >0.96 sen/spe	0.4	0.398	0.494	0.2	0.408	0.2	0	0.094	0.614	0.992
Proportion of simulations with >0.95 sen/spe	0.4	0.398	0.6	0.4	0.456	0.2	0	0.44	0.614	1
Proportion of simulations with >0.90 sen/spe	0.55	0.594	0.6	0.77	0.868	0.2	0.37	0.812	1	1
Proportion of simulations with >0.85 sen/spe	0.6	0.6	0.618	1	1	0.4	0.418	1	1	1
Proportion of simulations with >0.80 sen/spe	0.6	0.6	0.8	1	1	0.4	0.6	1	1	1
Proportion of simulations with >0.75 sen/spe	0.6	0.6	0.8	1	1	0.4	0.6	1	1	1
Symptoms used below										
mde_mi5_2	0	1	0	0	0	1	0	0	0	0
mde_mi9	0	1	0	0	0	1	0	0	0	0
dys_ma	0	1	0	0	0	1	0	0	0	0
man_ma1	22	28	0	0	0	28	21	1	0	0
man_ma2	18	23	0	0	0	23	16	1	0	1
man_ma3	401	450	500	500	500	351	500	500	500	500
man_mi1_1	138	117	99	0	0	85	163	82	8	16
man_mi1_2	137	119	99	0	0	84	160	86	13	12
man_mi2	330	403	400	185	28	141	383	417	276	129
man_mi3_1	122	96	98	0	0	83	150	68	6	9
man_mi3_2	121	98	98	0	0	88	138	75	9	7
man_mi4_1	132	109	100	0	0	91	165	69	6	10
man_mi4_2	144	106	100	0	0	93	159	76	8	14
man_mi5	344	404	400	185	28	145	382	419	293	122
man_mi6_1	125	102	106	0	0	92	159	60	6	16
man_mi6_2	130	110	106	0	0	88	166	63	14	15
man_mi7	347	412	400	185	28	145	383	420	284	140

In summary, when at least 92% sensitivities and specificities were acceptable for approximating the diagnoses, Table 8 shows the minimal sensitivities and specificities and the input symptoms required for approximation by assumed symptom prevalence and correlation. With low symptom prevalence (i.e., 0.05 or 0.1) and low correlations between symptoms (i.e., 0 or 0.1), it was less likely to approximate the diagnoses using fewer input symptoms to obtain sufficient sensitivities and specificities.

**Table 8:** Minimal sensitivities and specificities for approximating the diagnoses by assumed symptom prevalence and correlation. dys_ma = Depressed mood most of the day for more days than not, for at least two years; man_ma3 = Irritable mood, lasting at least one week; man_mi2 = Decreased need for sleep (e.g., feels rested after only three hours of sleep); man_mi5 = Distractibility (i.e., attention too easily drawn to unimportant or irrelevant external stimuli); man_mi7 = Excessive involvement in pleasurable activities that have a high potential for painful consequences (e.g., engaging in unrestrained buying sprees, sexual indiscretions, or foolish business investments)"; mde_ma1 = Depressed mood for more than two weeks; mde_ma2 = Loss of interest or pleasure in daily activities for more than two weeks according to the Diagnostic and Statistical Manual of Mental Disorders, 4th edition, text revision (DSM-IV-TR) in Table 1. Blank cells = 92% or higher sensitivities and specificities could not be achieved in all of the 100 simulations, given assumed symptom prevalence and correlations. The percentages in the cells refer to the minimal sensitivities and specificities that could be achieved given the prevalence and correlations of input symptoms. The N in the cells refer to the number of input symptoms required for approximation.

Diagnoses	Matrix of assumed symptom prevalence and correlations					
Major depressive episodes	Assumed correlations	Assumed prevalence				
		0.05	0.1	0.3	0.5	0.7
	0				99%, N=2 (mde_ma1 and mde_ma2)	100%, N=2 (mde_ma1 and mde_ma2)
	0.1			96%, N=2 (mde_ma1 and mde_ma2)	98%, N=2 (mde_ma1 and mde_ma2)	99%, N=2 (mde_ma1 and mde_ma2)
	0.4		99%, N=2 (mde_ma1 and mde_ma2)	98%, N=2 (mde_ma1 and mde_ma2)	98%, N=2 (mde_ma1 and mde_ma2)	99%, N=2 (mde_ma1 and mde_ma2)
	0.7	99%, N=2 (mde_ma1 and mde_ma2)	99%, N=2 (mde_ma1 and mde_ma2)	99%, N=2 (mde_ma1 and mde_ma2)	99%, N=2 (mde_ma1 and mde_ma2)	99%, N=2 (mde_ma1 and mde_ma2)
	0.9	100%, N=2 (mde_ma1 and mde_ma2)	99%, N=2 (mde_ma1 and mde_ma2)	99%, N=2 (mde_ma1 and mde_ma2)	99%, N=2 (mde_ma1 and mde_ma2)	99%, N=2 (mde_ma1 and mde_ma2)
Dysthymic disorder	Assumed correlations	Assumed prevalence				
		0.05	0.1	0.3	0.5	0.7
	0			92%, N=1 (dys_ma)	98%, N=1 (dys_ma)	99%, N=1 (dys_ma)
	0.1				96%, N=1 (dys_ma)	98%, N=1 (dys_ma)
	0.4				95%, N=1 (dys_ma)	95%, N=1 (dys_ma)
	0.7			97%, N=1 (dys_ma)	97%, N=1 (dys_ma)	96%, N=1 (dys_ma)
	0.9	99%, N=1 (dys_ma)	99%, N=1 (dys_ma)	98%, N=1 (dys_ma)	98%, N=1 (dys_ma)	98%, N=1 (dys_ma)
Manic episodes	Assumed correlations	Assumed prevalence				
		0.05	0.1	0.3	0.5	0.7
	0					97%, N=1 (man_ma3)
	0.1					93%, N=1 (man_ma3)
	0.4			95%, N=4 (man_ma3, man_mi2, man_mi5, and man_mi7)	95%, N=4 (man_ma3, man_mi2, man_mi5, and man_mi7)	
	0.7			98%, N=4 (man_ma3, man_mi2, man_mi5, and man_mi7)	93%, N=1 (man_ma3)	92%, N=4 (man_ma3, man_mi2, man_mi5, and man_mi7)
	0.9			97%, N=1 (man_ma3)	96%, N=1 (man_ma3)	95%, N=1 (man_ma3)

## Discussion

There might be opportunities to adjust the diagnostic criteria of three mental illnesses for local contexts: major depressive episodes, dysthymic disorder, and manic episodes based on the DSM-IV-TR diagnostic criteria. This is because some of the input symptoms have been implicitly given little weight and have contributed little to their diagnoses under certain circumstances [1]. The diagnostic criteria of the three mental illnesses are overcomplicated and assign more weight to some of the input symptoms [2]. For example, the third input symptom in the major criteria for the diagnosis of manic episodes, “irritable mood lasting at least one week,” can explain 48.3% of the variances of the diagnosis of manic episodes in some situations [2]. This study aims to understand how to take advantage of the differences in symptom importance in clinical practice.

Using decision trees, a population is separated into two groups by symptoms [8]. A symptom that best distinguishes the diseased from those not diseased is used first and placed on top of a decision tree [8]. This step is repeated until the diagnostic accuracy cannot be further improved [8]. This approach is similar to a clinical scenario in which a clinician screens patients based on the importance of symptoms, rather than checking all symptoms or items in a comprehensive list before making a diagnosis. We have used decision trees to diagnose three mental illnesses in this study. Using input symptoms of their own, we continued to confirm that the diagnostic criteria of mental illnesses assigned much more weight to some of the input symptoms than the other input symptoms.

In the combinations of (1) symptom prevalence close to 0.7 and between-symptom correlations close to 0 and (2) symptom prevalence close to 0.05 and between-symptom correlations close to 0.9, it takes only two input symptoms (mde_ma1: Depressed mood for more than two weeks, and mde_ma2: Loss of interest or pleasure in daily activities for more than two weeks) to diagnose major depressive episodes with 100% sensitivities and specificities. These scenarios can be plausible because the prevalence of major depressive disorder has been estimated to be 4.7% globally [12]. Among adults who have recovered from depression, 67% can have recurrence within 10 years [13]. It may be possible to detect new cases quickly or follow up recurrence by screening for two symptoms only, if the contexts fall into the above scenarios.

Opportunities to simplify diagnostic criteria

In addition to achieving 100% of sensitivities and specificities, if it is acceptable to have at least 92% sensitivities and specificities for approximating the diagnoses, there are more opportunities to prioritize certain input symptoms and simplify the diagnostic criteria. For major depressive episodes, with symptom prevalence close to 0.5 or 0.7 or between-symptom correlations close to 0.7 or 0.9, it took two input symptoms (mde_ma1: Depressed mood for more than two weeks, and mde_ma2: Loss of interest or pleasure in daily activities for more than two weeks) to approximating the diagnosis with at least 98% sensitivities or specificities. With symptom prevalence close to 0.1 or 0.3 and between-symptom correlations close to 0.4, it took the same two of the 15 input symptoms to approximate the diagnosis. For dysthymic disorder, with symptom prevalence close to 0.5 or 0.7, it took one input symptom (dys_ma: Depressed mood most of the day for more days than not, for at least two years) for approximating the diagnosis with at least 95% sensitivities or specificities. With between-symptom correlations close to 0.9, it took the same one input symptom for approximating the diagnosis with at least 98% sensitivities or specificities. In the real world, the global prevalence of depressive symptoms can be 34% and 47% among adolescents [13] and homeless people [14], respectively. With the prevalence of depressive symptoms higher than 30%, there may lie opportunities to simplify the diagnostic or screening approaches and quickly identify cases of major depressive episodes and dysthymic disorder.

For manic episodes, with symptom prevalence close to 0.3, 0.5, or 0.7 and correlations between input symptoms close to 0.7 or 0.9, it takes at most four input symptoms (man_ma3: Irritable mood, lasting at least one week, man_mi2: Decreased need for sleep, man_mi5: Distractibility, and man_mi7: Excessive involvement in pleasurable activities that have a high potential for painful consequences) to approximate the diagnosis with at least 92% sensitivities or specificities. In the real world, the lifetime prevalence of manic episodes can be as high as 7.5% among Brazilian adults aged 18-24 years [15]. Among adults aged 50 years and over, the prevalence of manic episodes has been estimated to be 6%, and the prevalence of late-onset manic episodes could be as high as 44% of inpatients with bipolar disorder [16]. The diagnostic criteria of manic episodes might have the potential to be simplified if sufficiently high symptom prevalence and correlations can be confirmed in the real world.

Input symptoms for approximating diagnoses

Similar to previous findings [1], we found that some input symptoms better approximate diagnoses than other input symptoms. For example, two symptoms (mde_ma1: Depressed mood for more than two weeks, and mde_ma2: Loss of interest or pleasure in daily activities for more than two weeks) could approximate the diagnosis of major depressive episodes with 100% sensitivities and specificities under some circumstances. Some input symptoms have been consistently used to approximate diagnoses, while some have rarely been used throughout the simulations. For example, the symptom in the major criteria (dys_ma: Depressed mood most of the day for more days than not, for at least two years) has been used to approximate the diagnosis of dysthymic disorder with at least 92% sensitivities or specificities under some combinations of symptom prevalence and correlations. This is due to multiple factors: design of the diagnostic criteria, prevalence of input symptoms, and correlations between input symptoms. The DSM-IV-TR groups symptoms into major and minor [1]. This grouping has been well received. Many regard the input symptoms in the major category as more important than those in the minor category [2]. This view can be supported by the fact that all of the input symptoms in the major criteria are needed for the diagnosis of major depressive episodes and dysthymic disorder in all eligible simulations. However, two of the input symptoms in the major criteria for the diagnosis of manic episodes (man_ma1: Elevated mood, lasting at least one week, and man_ma2: Expansive mood, lasting at least one week) are used less often than the symptoms in the minor criteria for approximating the diagnosis. The diagnostic criteria of the manic episodes have assigned much weight to the third input symptom in the major criteria (man_ma3: Irritable mood, lasting at least one week) and little to the first two. In clinical practice, questioning patients for symptoms that contribute little to the diagnosis or treatment plan can impose unnecessary time costs and psychological stresses on patients. We suggest further review of the design of the diagnostic criteria of manic episodes.

The importance of the input symptoms in the minor criteria diminishes with higher symptom prevalence or higher correlations between symptoms, especially for major depressive episodes and dysthymic disorder. With input symptom prevalence reaching 0.5 or 0.7, all input symptoms in the minor criteria are not required to achieve more than 95% sensitivities and specificities. This diagnostic accuracy is better than approximating diagnoses using specialized tools or questionnaires [4,17]. We think the input symptoms in the major criteria have the potential for timely and accurate monitoring of the three mood disorders.

Interactions between diagnoses

The three diagnoses can interact with each other due to the design of the diagnostic criteria and randomness. The diagnoses of major depressive episodes and dysthymic disorder share six input symptoms (mde_mi4_1: Insomnia, mde_mi4_2: Sleeping too much, mde_mi6_1: Fatigue, mde_mi6_2: Loss of energy, mde_mi8_1: Diminished ability to think or concentrate, and mde_mi8_2: Difficulty making decisions). This inevitably leads to sharing these symptoms in decision trees, especially when symptom prevalence and between-symptom correlations are less than 0.5. With symptom prevalence close to 0.05 or between-symptom correlations close to 0.1, there are times when the diagnosis of dysthymic disorder can be used to diagnose major depressive disorder. In a recent version of DSM, DSM-5, dysthymic disorder has been merged with chronic major depressive disorder [6].

Moreover, randomness may also be a factor. Sometimes the input symptoms of manic episodes are used in the decision trees to diagnose major depressive episodes, and vice versa. Even though the input symptoms of major depressive episodes are set up to be uncorrelated with those of manic episodes in the simulations. One reason to observe the occasional use of another diagnoses’ input symptoms for approximation may be related to the fact that the diagnoses of major depressive episodes and manic episodes both require more input symptoms than dysthymic disorder. However, this interaction may potentially be avoidable. Adopting the design of the diagnostic criteria of dysthymic disorder may be an approach to avoid unnecessary interactions between diagnoses.

Research implications

Our findings have major research implications. First, based on the findings in this study, we need to systematically collect two epidemiological measures of psychiatric symptoms: prevalence and correlations. By simulating the parameters that we learn from real-world data, we will be able to understand how to simplify the diagnostic criteria of mental illnesses for various clinical settings. We continue to investigate how to improve the diagnostic process and the diagnostic criteria for mental illnesses.

Second, mental health screening tools can be simplified to increase accessibility, improve efficiency, and even enhance diagnostic accuracy. In Table 8, the diagnostic accuracy of four or fewer input symptoms can be 92% or higher under certain combinations of symptom prevalence and correlations. This finding can help various online mental health screening tools [18,19] and mental health screening tools used in primary care [20] to maintain diagnostic accuracy and simplify the screening process by focusing on the symptoms or questions with the highest diagnostic accuracy. Previous research has shown that the first step to simplify diagnostic criteria is to select symptoms or questions with high diagnostic accuracy [7]. Then, the correlations between symptoms or questions can lead to improvements in diagnostic accuracy upon single symptoms or questions [7]. Based on published evidence and our findings, the symptoms used for screening may be prioritized, and fewer screening questions may help improve access to these online screening tools.

Third, our findings suggest that clinical practice to diagnose mental illnesses can be improved using interviews that look into the symptoms that have been implicitly assigned higher weights in diagnostic criteria. These symptoms are those that appear more often in decision tree models in Tables 3-7. For example, the third symptom in the major criteria for the diagnosis of manic episodes (man_ma3: Irritable mood, lasting at least one week) has been used in all simulated populations with any cases of manic episodes. Three of 11 input symptoms in the minor criteria for the diagnosis of manic episodes (man_mi2: Decreased need for sleep, man_mi5: Distractibility, man_mi7: Excessive involvement in pleasurable activities that have a high potential for painful consequences) have been used more often than the other symptoms in the minor criteria, if the other symptoms have been used at all. All of the input symptoms in the major criteria for the diagnosis of major depressive episodes and dysthymic disorder have been used in all simulations. However, one (mde_mi9: Recurrent thoughts of death) and two (dys_mi4: Low self-esteem, dys_mi6: Feelings of hopelessness) of the 13 and 10 input symptoms in the minor criteria for the diagnosis of major depressive episodes and dysthymic disorder, respectively, have been used more often than the other input symptoms in the minor criteria of their respective diagnoses. We are testing other combinations of symptom prevalence and correlation to make recommendations about how to prioritize these symptoms that have been implicitly assigned more weight in clinical practice.

Finally, we think the DSM authors need to align the diagnostic criteria with more epidemiological evidence. There is currently a lack of real-world data on psychiatric symptoms to support existing diagnostic criteria [2]. There may be a lack of causal relationship between mental illness diagnoses and their symptoms [3]. In other words, some psychiatric symptoms may not be caused by the diagnoses that these symptoms are used to diagnose [3]. Except for the third symptom (man_ma3: Irritable mood, lasting at least one week), the symptoms in the major criteria for the diagnosis of manic episodes are, in fact, used less often than those in the minor criteria in this study. We think there is a major gap in the evidence to support the current approach of using symptoms to diagnose mental illnesses. We are actively collecting epidemiological evidence and determining the strength of association between psychiatric symptoms and mental illness diagnoses.

Policy implications

There is an important policy implication: focusing on local contexts and the impact on symptom prevalence and correlations. Our findings suggest that clinicians and researchers need to understand local contexts and their impact on psychiatric symptom prevalence and correlations in a population to best use the diagnostic criteria. The DSM system has identified several culture-bound syndromes that are unique to certain cultures [21]. Since DSM-IV, cultural contexts have been considered, and an outline for cultural formulation has been published to guide culturally sensitive practice [22]. A cultural formulation interview tool has been developed and used to assist clinical practice and better understand the contexts of psychiatric symptoms [22]. The main themes in the cultural formulation interview include the following: cultural perceptions of cause, context, and support; role of cultural identity; cultural factors affecting self-coping; and past help-seeking [22]. This tool has been useful for improving communication and facilitating treatment planning [23]. However, tools like this may not be used often, and clinicians have reported using the cultural formulation interview on a limited number of occasions [24]. For symptom prevalence and correlations, we think current guidance is insufficient for us to understand how local contexts, such as cultural contexts, may influence how psychiatric symptoms are reported and documented separately or together. Moreover, there is an insufficient documentation of psychiatric symptoms in clinical practice or in research settings [1,2].

We think policies that aim to improve the documentation of psychiatric symptoms and contextual factors are needed. There are several policy options to improve the documentation of psychiatric symptoms and local contexts, such as raising the awareness of the importance of symptom recording, making the documentation of psychiatric symptoms a central part of medical records, improving the acces to tools, such as cultural formulation interview, for clinicians to assess contexts that may influence symptom prevalence and correlations, introducing automation tools to record psychiatric symptoms during consultation and follow-up. With our evidence on the role of individual symptoms, we think several changes to medical education and clinical practice are needed.

Limitations

This study adopts simulation models from published studies [1,7] and an analytical approach that may align with clinical practice well. However, there are several limitations to this simulation study. Without real-world data on symptom prevalence and correlations [1], the only option is to simulate symptoms of various prevalence rates and correlations. Some of the assumed values may not be realistic. We assumed similar prevalence and correlations for all input symptoms, but the input symptoms of manic episodes did not correlate with those of the other two diagnoses. In the real world, the input symptoms for mental illnesses are likely to differ in prevalence and do not correlate with each other uniformly.

## Conclusions

There may be opportunities to simplify the diagnostic criteria of major depressive episodes because only two input symptoms (mde_ma1: Depressed mood for more than two weeks, and mde_ma2: Loss of interest or pleasure in daily activities for more than two weeks) are required to diagnose this condition with 100% sensitivities and specificities in simulations with certain combinations of symptom prevalence or symptom correlations. There may lie opportunities to screen or follow up the diagnosis of major depressive episodes, dysthymic disorder, and manic episodes using fewer input symptoms with at least 92% sensitivities and specificities. This is because the diagnostic criteria of these diagnoses have been designed so complicated that clinicians and researchers are unaware of the effects of the differential weights assigned to input symptoms. Some input symptoms, either considered major or minor by the DSM-IV-TR criteria, may not contribute to their diagnoses at all in some combinations of symptom prevalence and correlations. In clinical scenarios where there are difficulties in assessing all input symptoms, screening or following up input symptoms that matter the most for the diagnosis of mental illnesses may be a superior option. However, in the real world, there is a lack of data on psychiatric symptoms and how they lead to mental illness diagnoses. We did not identify interventions or screening programs taking advantage of input symptoms that have superior diagnostic accuracy. These opportunities have the potential to facilitate mental illness diagnosis and screening.
